# Inhibition of LIFR Blocks Adiposity-Driven Endometrioid Endometrial Cancer Growth

**DOI:** 10.3390/cancers14215400

**Published:** 2022-11-02

**Authors:** Logan Blankenship, Uday P. Pratap, Xue Yang, Zexuan Liu, Kristin A. Altwegg, Bindu Santhamma, Kumaraguruparan Ramasamy, Swapna Konda, Yidong Chen, Zhao Lai, Siyuan Zheng, Gangadhara R. Sareddy, Philip T. Valente, Edward R. Kost, Hareesh B. Nair, Rajeshwar R. Tekmal, Ratna K. Vadlamudi, Suryavathi Viswanadhapalli

**Affiliations:** 1 Department of Obstetrics and Gynecology, University of Texas Health San Antonio, San Antonio, TX 78229, USA; 2 Department of Obstetrics and Gynecology, Second Xiangya Hospital, Central South University, Changsha 410011, China; 3 Department of Oncology, Xiangya Hospital, Central South University, Changsha 410008, China; 4 Mays Cancer Center, University of Texas Health San Antonio, San Antonio, TX 78229, USA; 5 Evestra, Inc., San Antonio, TX 78245, USA; 6 Greehey Children’s Cancer Research Institute, University of Texas Health San Antonio, San Antonio, TX 78229, USA; 7 Department of Population Health Sciences, University of Texas Health San Antonio, San Antonio, TX 78229, USA; 8 Department of Molecular Medicine, University of Texas Health San Antonio, San Antonio, TX 78229, USA; 9 Audie L. Murphy Division, South Texas Veterans Health Care System, San Antonio, TX 78229, USA

**Keywords:** leukemia inhibitory factor, leukemia inhibitory factor receptor, endometrioid endometrial cancer, obesity, EC359, RNA-seq

## Abstract

**Simple Summary:**

In this study, we utilized global RNA-seq to elucidate the molecular mechanisms by which obese conditions promote progression of endometrioid endometrial cancer (EEC). Our results suggest that obese conditions upregulate LIF/LIFR signaling, and EEC tumors collected from obese patients have high levels of LIF. Mechanistic studies suggest that LIF/LIFR signaling plays an important role in obesity-driven EEC progression and the LIFR inhibitor, EC359, has the potential to suppress the tumor progression driven by increased adiposity found in obese patients.

**Abstract:**

Endometrial cancer (EC) is the fourth most common cancer in women, and half of the endometrioid EC (EEC) cases are attributable to obesity. However, the underlying mechanism(s) of obesity-driven EEC remain(s) unclear. In this study, we examined whether LIF signaling plays a role in the obesity-driven progression of EEC. RNA-seq analysis of EEC cells stimulated by adipose conditioned medium (ADP-CM) showed upregulation of LIF/LIFR-mediated signaling pathways including JAK/STAT and interleukin pathways. Immunohistochemistry analysis of normal and EEC tissues collected from obese patients revealed that LIF expression is upregulated in EEC tissues compared to the normal endometrium. Treatment of both primary and established EEC cells with ADP-CM increased the expression of LIF and its receptor LIFR and enhanced proliferation of EEC cells. Treatment of EEC cells with the LIFR inhibitor EC359 abolished ADP-CM induced colony formation andcell viability and decreased growth of EEC organoids. Mechanistic studies using Western blotting, RT-qPCR and reporter assays confirmed that ADP-CM activated LIF/LIFR downstream signaling, which can be effectively attenuated by the addition of EC359. In xenograft assays, co-implantation of adipocytes significantly enhanced EEC xenograft tumor growth. Further, treatment with EC359 significantly attenuated adipocyte-induced EEC progression in vivo. Collectively, our data support the premise that LIF/LIFR signaling plays an important role in obesity-driven EEC progression and the LIFR inhibitor EC359 has the potential to suppress adipocyte-driven tumor progression.

## 1. Introduction

While endometrial cancer (EC) occurs most commonly in older women, the mortality rate and incidence are exponentially increasing in women under 40 years of age [1,2]. Estrogen (E2, 17-β-estradiol) signaling plays an important role in the progression of the EEC subtype of EC [3], and unopposed E2 exposure is implicated as a major risk factor for EEC [4]. Despite the promising results of progestins, hormone therapy confers moderate benefits on EC patients with a recurrence rate of approximately 50% [5]. There is an unmet need for the development of new targeted therapies that complement existing EC-directed therapies.

More than half of EC cases are attributable to obesity [6,7]. Body mass index (BMI) represents a major modifiable risk factor; approximately half of all EC cases in postmenopausal women are attributable to being overweight or obese [8]. Visceral adipose tissue is a significant and reliable prognostic indicator for EC prognosis [9]. The mechanisms by which obesity contribute to the pathogenesis of EC are poorly understood.

Obesity signaling enhances E2 biosynthesis [10] and E2 function as a potent inducer of leukemia inhibitory factor (LIF) [11,12,13], which mediate its signaling using a receptor complex comprising LIFR and glycoprotein 130 (gp130) [14]. JAK/STAT3 functions as an immediate effector while MAPK, AKT and mTOR function as further downstream effectors of the LIF/LIFR complex [15]. The role of LIF/LIFR signaling in obesity-mediated EEC progression remains unclear and is the focus of this study.

Here, using global RNA-seq analyses, we found that obesity conditions promote activation of IL6 cytokine and JAK/STAT3 signaling. EEC tumors from patients with a high BMI have increased levels of LIF expression. Obese conditions promote EEC proliferation and activation of LIF/LIFR signaling. Treatment with the LIFR inhibitor EC359 abolished obesity-mediated increases in LIFR signaling in vitro and EEC progression in vivo.

## 2. Materials and Methods

### 2.1. Cell Culture and Reagents

Human EC cell lines HEC-1-A, AN3 CA and RL95-2 were purchased from the American Type Culture Collection (ATCC, Manassas, VA, USA) and cultured as described [16]. Ishikawa cells were purchased from Sigma (Millipore Sigma, St. Louis, MO, USA). All model cells were free of mycoplasma contamination, and identity was confirmed using short tandem repeat polymorphism analysis (STR). EC359 was synthesized as described [17].

### 2.2. Primary EC Cells

Primary EC cells were established from patient-derived EC tissues using a University of Texas Health San Antonio (UTHSA) Institutional Review Board-approved protocol. These specimens were de-identified; both the PI and research staff did not have access to clinical linkers or codes. All cell lines were maintained in a humidified chamber with 5% CO_2_ at 37 °C. All the methods involving human tissues were conducted in accordance with the Declaration of Helsinki and the standards defined by UTHSA Institutional Review Board.

### 2.3. Human Adipose Tissue Samples

The University of Texas Health San Antonio (UTHSA) Institutional Review Board approved the protocol to collect the human adipose tissue. The visceral adipose tissues were collected in DMEM medium from human female patients with BMI > 30 kg/m^2^. Consent from patients was obtained as per approved guidelines.

### 2.4. Isolation of Mature Adipocytes from Human Adipose Tissue

The primary adipocytes (ADPs) were isolated from human adipose tissue as described previously [18]. Briefly, adipose tissue was minced, washed with DPBS and digested in 1 mg/mL Collagenase (type 1) solution for 1 h at 37 °C with gentle shaking. The digestion mixture was passed through a 100 µm cell strainer (BD Biosciences) and centrifuged at 800 rpm for 5 min. The upper floating layer containing the mature adipocytes was collected, washed and cultured in DMEM. After 48 h, the culture medium containing primary adipocytes was passed through a syringe filter (0.22 µm, Millipore) to collect adipocyte-conditioned medium (ADP-CM). Basal DMEM medium as serum-free medium (SFM) and 50% (*v*/*v*) ADP-CM were used in all in vitro experiments.

### 2.5. In Vitro Co-Culture Model

For co-culture, ADPs were seeded on an insert of a Boyden chamber. In the bottom well, endometrial cancer cells were seeded for the co-culture experiment. Control wells contained the insert without ADPs. Co-cultures were carried out in triplicate for 24 h, followed by lysis of endometrial cancer cells for protein and RNA isolation.

### 2.6. Cell Viability and Clonogenic Assays

The effect of EC359 treatment on adipose-induced cell viability of EEC cells was assessed after 5 days of treatment using MTT cell viability assay as previously described [17]. For clonogenic assays, EEC cells (500 cells/well) were plated in 6-well plates in triplicate treated with vehicle or ADP-CM or ADP-CM+EC359 for 5 days, and colonies that contained ≥50 cells were counted after 2 weeks.

### 2.7. Western Blotting and RT-qPCR

Cells were lysed using RIPA buffer, and Western blotting was performed as previously described [16,17]. The phosphorylation-specific antibodies (p-p70S6K(T389), p70S6K, p-Akt(S473), Akt, p-mTOR(S2448), mTOR, p-S6(S235/236), S6, p-ERK1/2, ERK, p-STAT3(Y705), and STAT3), were obtained from Cell Signaling Technology (Beverly, MA, USA). LIF and LIFR antibodies were purchased from Santa Cruz Biotechnology (Dallas, TX, USA). Anti β-actin antibody was obtained from Sigma. RT-qPCR was carried out using High-Capacity cDNA Reverse Transcription Kit and PowerUp SYBR Green master mix (Applied Biosystems) on a CFX96 Real-Time PCR system. Primer sequences are included in Supplementary Table S1. Original blots see Supplementary Figure S1.

### 2.8. Reporter Gene Assays

STAT3-luc assays were carried out as previously described [16]. Briefly, STAT3-luc reporter cells were serum-starved for 24 h and treated with ADP-CM or ADP-CM + EC359 for 24 h. Reporter activity was measured in luminometer using the dual-luciferase reporter assay system (Promega, Madison, WI, USA).

### 2.9. RNA-seq Analysis

The effect of ADP-CM on the global transcriptome was determined by RNA sequencing. Total RNA was isolated using RNeasy mini kit (Qiagen, Valencia, CA, USA), and RNA sequencing was performed by the genome sequencing core facility at UT Health San Antonio. Sequence data were aligned to human genome (UCSC hg19) with TopHat2 [19]. Genes were annotated by NCBI RefSeq and quantified by HTSeq [20]. Differentially expressed genes were identified using DESeq [21]. For pathway analyses, genes with fold change > 1.5 and multiple-test-adjusted *p*-value < 0.01 were used. Gene Set Enrichment Analysis (GSEA) was performed on 13 February 2021 using published protocol (http://www.gsea-msigdb.org/gsea/index.jsp, accessed on 21 September 2022) [22].

### 2.10. In Vivo Xenograft Models

All animal experiments were performed using approved UT Health San Antonio IACUC protocol and guidelines. Female SCID mice (n = 5 tumors/group) were injected with RL95-2 and mature ADP cells (1 × 10^6^) mixed with equal volume of growth-factor-reduced Matrigel. After establishment of tumors, mice were randomized into control (0.3% hydroxy propyl cellulose) group and the treatment group (EC359-5 mg/kg/bw/day/ip). Body weight and tumor growth were measured at 3-4-day intervals. Tumor volume was calculated using a formula: tumor volume = 1/2(L × W^2^), where L is the longitudinal diameter and W is the transverse diameter. At the end of the experiment, mice were euthanized, and tumors were collected and processed for histological studies.

### 2.11. Tissue Microarray (TMA) and Immunohistochemistry (IHC)

EEC TMAs were obtained from the UTHSA Ob-Gyn Tissue Core and used for this study. The Core has a collection of both normal and cancerous endometrial tissues with BMIs ranging from 30 to 68 kg/m^2^ (Supplementary Table S2). Immunohistochemistry (IHC) was performed as described [16]. TMAs were probed with the anti-LIF antibody, and tumor sections were incubated with anti-Ki67 antibody (Abcam, Cambridge, MA, USA) overnight at 4 °C. Secondary antibody incubation took place over 45 minutes at room temperature. IHC slides were developed using the DAB substrate and counterstained with hematoxylin (Vector Lab, Burlingame, CA, USA).

### 2.12. Patient-Derived Organoid (PDO) Studies

Patient-derived organoids (PDOs) were established, cultured and plated as previously described [16]. Organoids were treated with indicated concentration of EC359 in the presence or absence of ADP-CM. Cell viability was measured after 7 days of treatment using the CellTiter-Glo Assay kit and quantified using a GloMax^®^ Discover System (Promega, Madison, WI, USA).

### 2.13. Statistical Analyses

The unpaired Student’s t-test and one-way ANOVA were used to analyze the differences, and *p* < 0.05 was considered as significant. Statistical analyses were carried out using GraphPad Prism 9 software (San Diego, CA, USA).

## 3. Results

### 3.1. RNA-seq Analysis of EEC Cells Identified Unique Pathways Modulated by Adipose Conditions

**Figure 1:**
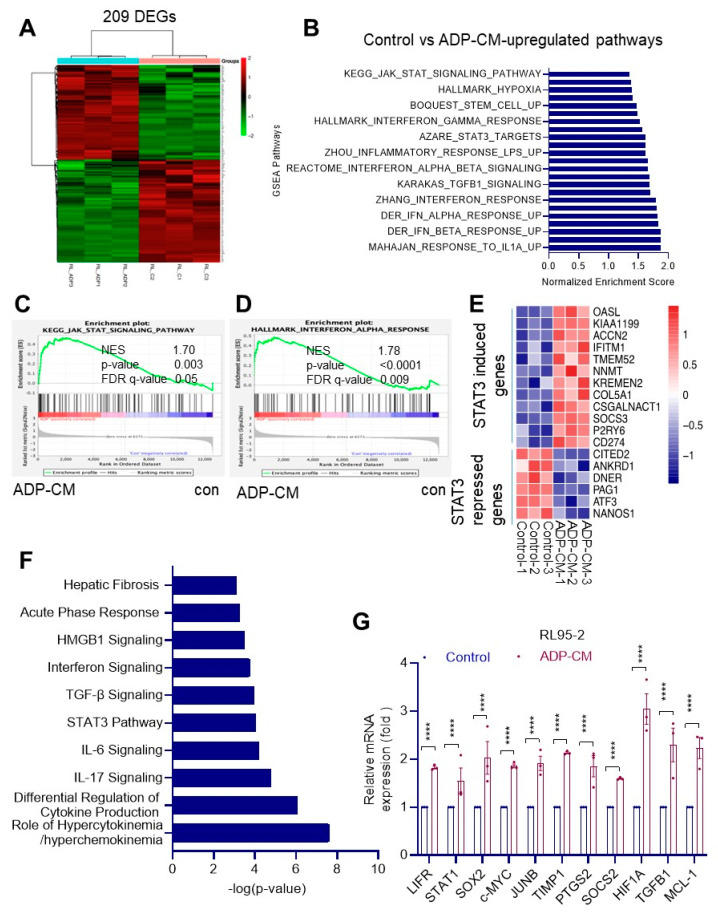
Global RNA-seq analysis of RL95-2 cells treated with ADP-CM identified unique pathways. (**A**) Heatmap showing differentially expressed genes upon ADP-CM treatment with |FC | > 1.5. (**B**–**D**) GSEA showing positively enriched pathways in ADP-CM-treated cells. GSEA plots show JAK-STAT pathway (**C**) and interferon alpha (**D**) response gene signatures were positively enriched in ADP-CM-treated group. (**E**) Heatmap shows the regulation of several known STAT3 target genes in ADP-CM-treated cells. (**F**) Ingenuity pathway analysis shows the top 10 pathways upregulated in ADP-CM-treated cells. (**G**) Validation of LIFR target genes by RT-qPCR. Data are represented as mean ± SE. **** *p* < 0.0001.

To better understand the mechanisms by which obese conditions stimulate EEC proliferation, we used a global RNA-seq approach. For this experiment, we used EEC cells cultured in the presence or absence of ADP-CM. A total of 209 genes were significantly changed by ADP-CM treatment compared to the control (Figure 1A). Analyses of ADP-CM-regulated genes using GSEA revealed a positive correlation with cytokine and STAT3 signaling pathways, stem cells, inflammation, TGFβ and the interferon signaling pathway (Figure 1B–D). Further analyses showed that ADP-CM increased the STAT3-induced genes and reduced the STAT3-repressed genes in EEC cells (Figure 1E). IPA analyses also confirmed that culturing of EEC cells in adipose-conditioned medium positively modulated several signaling pathways including IL6 and STAT3 (Figure 1F). RT-qPCR analysis of ADP-CM-treated samples showed increased expression of LIFR target genes compared to the control (Figure 1G).

### 3.2. EEC Tumors from High-BMI Cases Have Elevated LIF Expression

**Figure 2:**
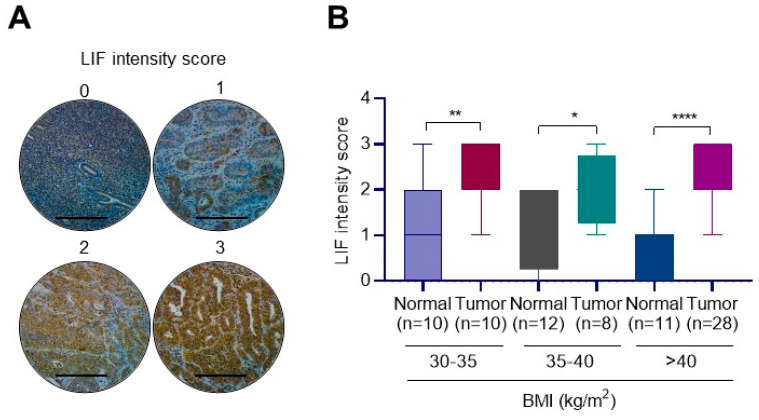
LIF is overexpressed in EEC. (**A**) Tissue microarray consisting of samples from patients with EEC (n = 46) and from individuals with normal endometrial tissue (n = 33) were evaluated for LIF expression, and representative IHC images are shown with LIF intensity score 0–3. (**B**) Quantitation of expression of LIF in normal and EEC tissue microarray is shown. Data are represented as mean ± SE. * *p* < 0.05; ** *p* < 0.01; **** *p* < 0.0001.

Earlier studies showed that increased BMI and obesity are strongly associated with EEC incidence and mortality [8]. Since our RNA-seq analyses identified that STAT3 and IL6 pathways are modulated by obese conditions, we examined whether obese conditions altered levels of LIF, an IL6 family of cytokine. We used tumor tissue arrays that contained normal (n = 33) and EEC (n = 46) tumors for IHC analyses using the LIF antibody. Results show that LIF expression from high-BMI cases was upregulated in EEC tumors compared to normal cases (Figure 2A,B; Supplementary Table S2).

### 3.3. Obese Conditions Enhance Cell Viability and LIF/LIFR Downstream Signaling

**Figure 3:**
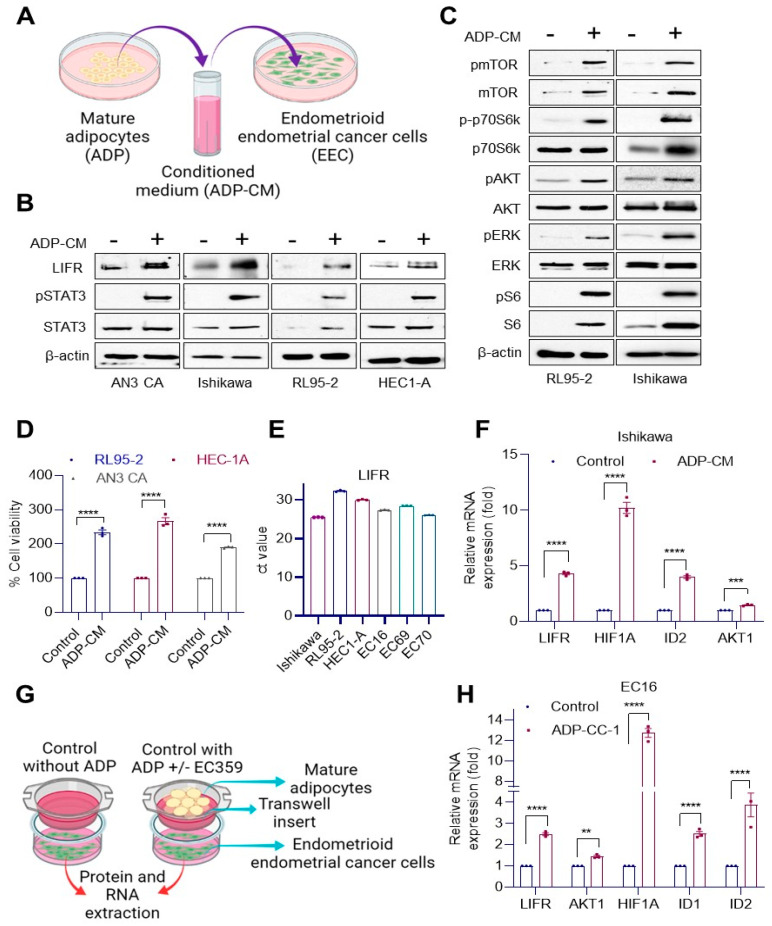
Adipocytes induced LIFR signaling in EEC cells. (**A**) Schematic of ADP-CM treatment of EEC cells. EEC cells were treated with ADP-CM for 24 h, and the expression of LIFR, pSTAT3, STAT3 (**B**) and LIFR downstream signaling (**C**) was determined by Western blotting. (**D**) Effect of adipose conditions on EEC cell proliferation was determined by MTT cell viability assay. (**E**) LIFR gene expression in established and primary EEC cells was analyzed by RT-qPCR. (**F**) Ishikawa cells were incubated with ADP-CM for 24 h, and the expression of LIFR target genes was analyzed by RT-qPCR. (**G**) Schematic of transwell co-culture system. (**H**) Adipocytes were indirectly co-cultured with primary EEC model cells (EC16) using a transwell culture system for 24 h, and the expression of LIFR target genes was analyzed by RT-qPCR. Data are represented as mean ± SE. ** *p* < 0.01; *** *p* < 0.001; **** *p* < 0.0001.

We then examined whether obese conditions promote LIF/LIFR signaling using two widely used models: (1) culturing of EEC cells in the presence or absence of conditioned medium (CM) collected from mature adipocytes (ADPs) (Figure 3A); (2) co-culture of EEC cells with adipocytes (Figure 3G). Western blotting analyses showed that ADP-CM increased the expression of LIFR in AN3 CA, Ishikawa, RL95-2 and HEC1-A cells (Figure 3B) and enhanced phosphorylation of STAT3, a downstream effector of LIFR (Figure 3B). Western blotting analysis also confirmed that ADP-CM enhances activation of several LIF/LIFR downstream effectors including mTOR, AKT, S6 and ERK kinases (Figure 3C). The addition of ADP-CM enhanced cell proliferation of EEC cells (Figure 3D). We validated the expression of LIFR in established and primary EEC cell lines by RT-qPCR analysis (Figure 3E). Further, RT-qPCR analyses confirmed that ADP-CM enhanced expression of known LIF/LIFR target genes (Figure 3F). Co-culture of primary EEC cells with adipocytes also showed increased expression of LIF/LIFR target genes (Figure 3H). Collectively, these results suggest that obese conditions enhance LIF/LIFR signaling activation in EEC.

### 3.4. LIFR Inhibitor, EC359, Reduced ADP-CM-Induced Cell Viability, Colony Formation and LIFR Downstream Signaling

**Figure 4:**
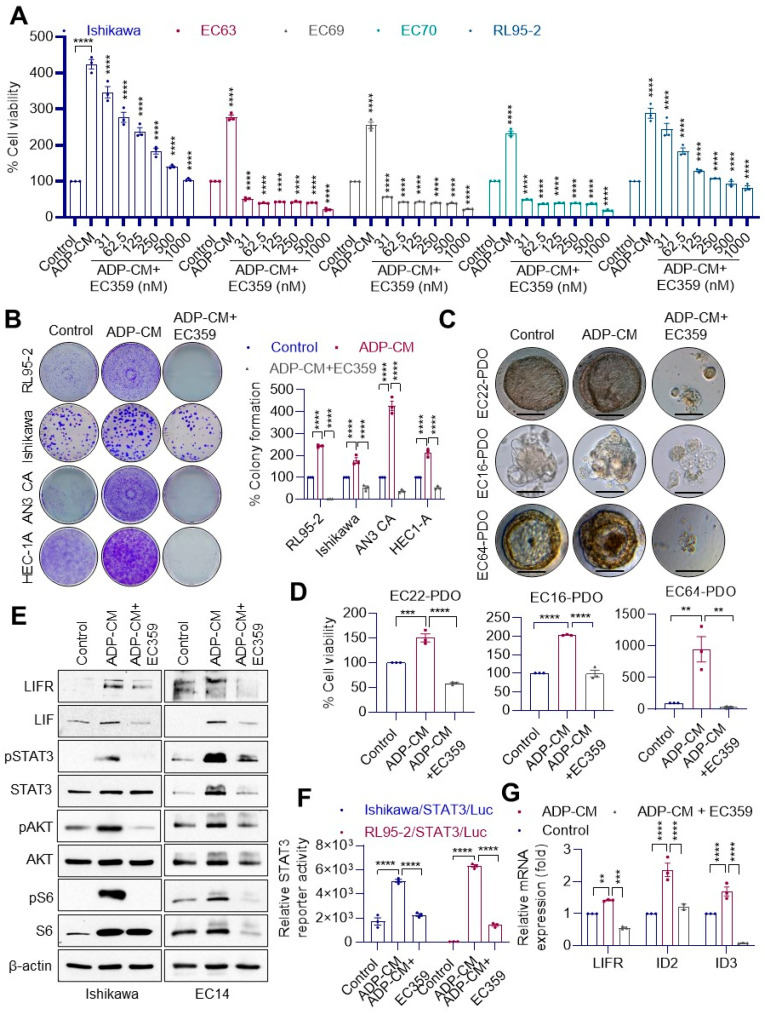
EC359 reduced adiposity-induced cell viability, colony formation and LIFR downstream signaling. (**A**) Established and primary endometrial cancer cells were incubated with ADP-CM with or without EC359 and cell proliferation was determined by MTT cell viability assay. (**B**) Effect of EC359 on adiposity-induced cell survival of EEC cells was measured using colony formation assays, and quantitation is shown on the right panel. (**C**) Representative images of PDOs cultured in ADP-CM in the presence or absence of EC359 are shown. (**D**) Effect of EC359 on adiposity-induced cell viability of organoids was measured using CellTiter-Glo 3D-Superior Cell Viability Assay. (**E**) Ishikawa and primary EEC (EC14) cells were treated with ADP-CM, and expression of LIFR and downstream signaling proteins were analyzed using Western blot analysis. (**F**) EEC cells stably expressing STAT3-luc were treated with ADP-CM in the presence or absence of EC359, and STAT3 reporter activity was measured after 24 h. (**G**) Primary EEC cells (EC16) incubated with ADP-CM with or without EC359, and the expression of LIFR target genes was analyzed by RT-qPCR. Data are represented as mean ± SE. ** *p* < 0.01; *** *p* < 0.001; **** *p* < 0.0001.

We examined whether the recently developed LIFR inhibitor, EC359, has the ability to block obesity-induced LIF/LIFR-mediated proliferation and signaling in EEC. The addition of EC359 decreased ADP-CM-induced cell viability in a dose-dependent manner (Figure 4A). Further, ADP-CM enhanced the colony formation ability of EEC cells that is abolished by EC359 treatment (Figure 4B). The addition of ADP-CM increased the viability of EEC organoids, which was attenuated by the addition of EC359 (Figure 4C,D). Western blotting analysis confirmed that ADP-CM increased the expression of LIF, LIFR and activated LIF/LIFR downstream signaling, which was effectively reduced with the addition of EC359 (Figure 4E). Using model cells that stably express the STAT3 reporter, we also confirmed that ADP-CM activated STAT3 reporter activity, which can be effectively decreased by the addition of EC359 (Figure 4F). RT-qPCR results also confirmed that EC359 effectively blocked the expression of LIFR target genes (Figure 4G). Collectively, these studies suggested that EC359 has the ability to block ADP-CM-mediated increases in LIF/LIFR signaling.

### 3.5. EC359 Reduced LIFR Downstream Signaling in EEC Cells Co-Cultured with Adipocytes

**Figure 5:**
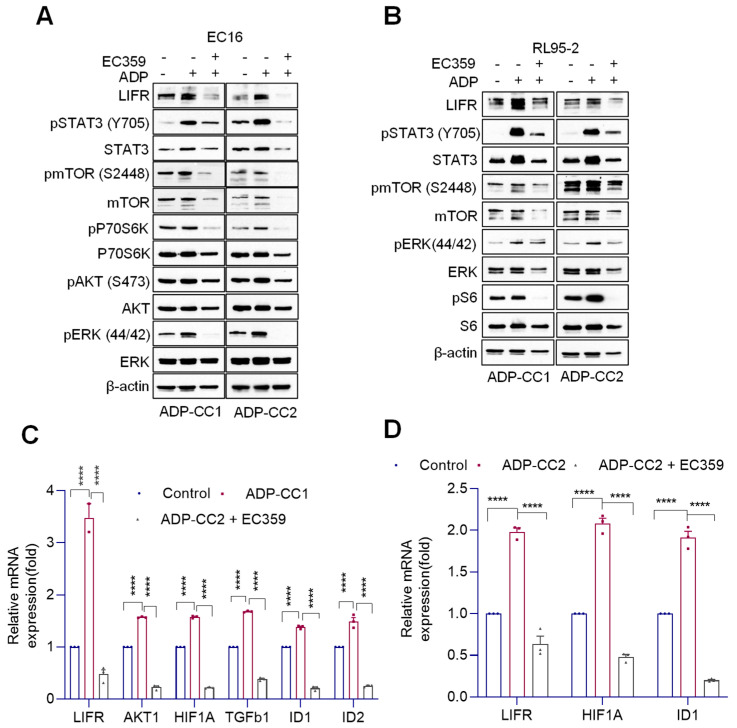
EC359 reduced LIFR downstream signaling in EEC cells co-cultured with adipocytes. (**A**,**B**), primary EEC (EC16) (**A**) and RL95-2 (**B**) cells were co-cultured with adipocytes (ADP-CC1 and ADP-CC2) for 24 h with or without EC359, and the expression of LIFR and downstream signaling proteins were analyzed using Western blot analysis. (**C**,**D**) Effect of EC359 treatment using RL95-2 cells co-cultured with ADP-CC1 (**C**) and ADP-CC2 (**D**) for 24 h on LIFR-targeted genes was measured using RT-qPCR. Data are represented as mean ± SE. **** *p* < 0.0001.

To test the potential of EC359 in blocking direct adipocyte-mediated enhanced LIFR signaling in EEC cells, we co-cultured primary human adipocytes (ADP-CC1 and ADP-CC2) obtained from the Ob/Gyn Core with primary EEC cell line EC16 (Figure 5A) or established EEC cell line RL95-2 (Figure 5B) in transwell inserts using a previously published protocol [23,24]. Western blotting results show that co-culture with adipocytes significantly enhanced LIFR expression; its downstream signaling in EEC model cells is effectively blocked by EC359 treatment (Figure 5A,B). Further, co-culture of adipocytes with EEC cells also activated LIFR target genes, and the activation was then blocked by EC359 treatment (Figure 5C,D). Collectively, the results support that EC359 has the potential to block adipocyte-mediated enhanced LIF/LIFR signaling.

### 3.6. Adipose Cells Promote EEC Progression, and Functional LIFR Signaling Is Needed for Optimal Growth of EEC In Vivo

**Figure 6:**
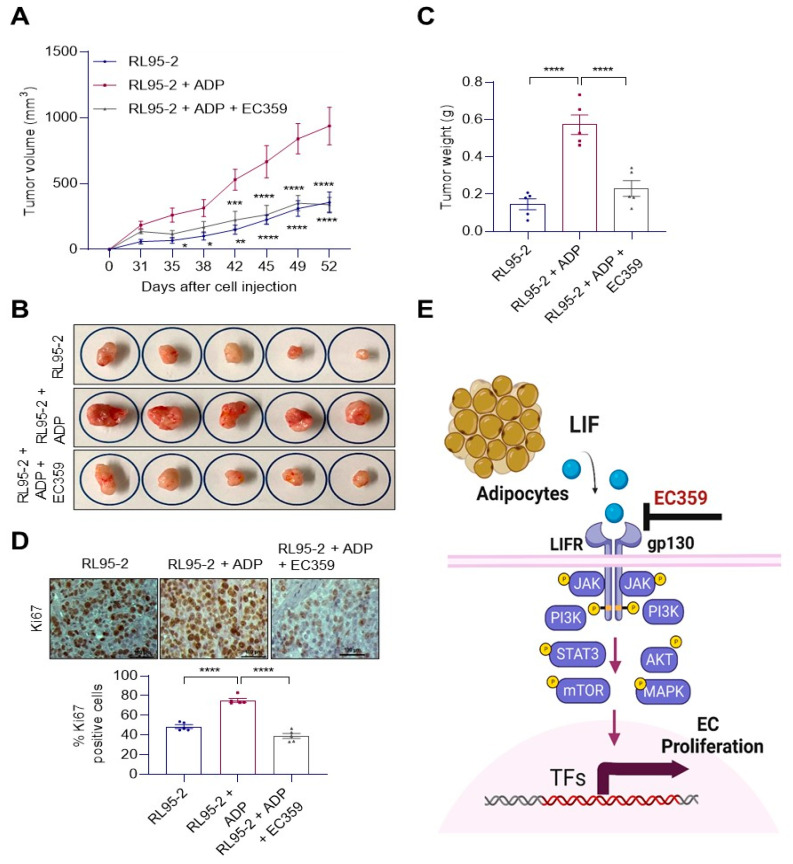
EC359 treatment inhibits adiposity-induced in vivo xenograft tumor growth. A-C, RL95-2 cells were injected along with mature adipocytes and treated with or without EC359. RL95-2 cells injected along with mature adipocytes served as a vehicle. Tumor volume was measured twice a week. Tumor volume (**A**), tumor images (**B**), tumor weights (**C**) and IHC of Ki67 (**D**) are shown. (**E**) Schematic representation: Obesity conditions activate LIF/LIFR signaling, and this promotes EC progression via activation of STAT3, mTOR, AKT, MAPK signaling. Data are represented as mean ± SE. * *p* < 0.05; ** *p* < 0.01; *** *p* < 0.001; **** *p* < 0.0001.

To confirm that adipocytes enhance growth of EEC cells in vivo, we co-implanted RL95-2 cells with adipose cells. RL95-2 cells alone or in combination with mature adipocytes were injected subcutaneously with growth-factor-reduced Matrigel into the flanks of 8-week-old female SCID mice using a published procedure [25]. As shown in Figure 6A–C, co-implantation of adipocytes significantly enhanced EEC xenograft tumor growth. Further, treatment with EC359 significantly attenuated adipocyte-induced EEC progression (Figure 6A–C). IHC analysis of xenograft tissues showed that co-implanted tumors had high levels of proliferation marker Ki67 compared to RL95-2 xenografts and EC359 treated xenografts showed reduced expression of Ki67 (Figure 6D) compared to co-implanted xenografts. These data support the premise that LIFR signaling plays an important role in adipocyte-driven EEC progression and EC359 has the potential to suppress the adipocyte-driven tumor progression (Figure 6E).

## 4. Discussion

The obesity epidemic is expected to increase EEC cases in the USA [6]. Adjuvant hormone therapy only confers a moderate benefit on EC patients with a recurrence rate of approximately 50% [5]. Targeted agents in clinical trials, such as bevacizumab, everolimus and metformin, despite showing moderate efficacy, did not led to durable remission in patients with recurrent or advanced EC [26,27,28]. Patients diagnosed with type I early-stage EC often have a favorable prognosis; however, those with advanced stage, recurrent or high-grade EC have worse outcomes [27]. Therefore, understanding the mechanisms that contribute obesity-driven EC is critical to discovering new therapeutic targets and to developing new therapies for EC.

Among all cancers, increased BMI and obesity are strongly associated with EC incidence and mortality [8]. BMI represents a major modifiable risk factor; approximately half of all EC cases in postmenopausal women are attributable to being overweight or obese [8]. Visceral adipose tissue is a significant and reliable prognostic indicator for EC prognosis [9]. More than half of ECs are currently attributable to obesity, which is an independent risk factor for EEC [29]. Using tumor tissues collected from high-BMI and low-BMI cases, we have demonstrated that tumors from high-BMI patients exhibit increased levels of LIF compared to low-BMI patients.

Cellular components of adipose tissue (adipocytes, preadipocytes and mesenchymal stem cells) are the predominant source of aromatase, the enzyme that facilitates the production of estrogen [30]. Obesity increases the local concentration of E2 levels, which directly promotes EEC proliferation [30]. The mechanisms by which obesity and adipose tissue contribute to the pathogenesis of EC are poorly understood. Our studies using global RNA-seq suggested that obese conditions aberrantly activate cytokines and Jak/STAT3 pathway. Mechanistic studies confirmed that obese conditions enhance the expression of LIF/LIFR and activate LIFR downstream signaling.

Tumors are shown to exhibit LIF/LIFR autocrine and paracrine signaling mechanisms [31,32,33]. LIF signaling promotes tumor cells crosstalk with fibroblasts and is implicated in pro-invasive activation of stromal fibroblasts [34]. Obese conditions promote high levels of leptin; leptin increases LIF and LIFR levels in endometrial cells [12]. Further, LIF is a commonly upregulated gene in carboplatin- and paclitaxel-resistant cells [35]. In this study, we found that obese conditions promote activation of LIF/LIFR signaling. These findings are in agreement with published studies and suggest that obese conditions have the potential to create LIF/LIFR autocrine signaling loops, leading to progression of EEC.

The LIF/LIFR axis is implicated in tumor growth and progression by acting on multiple aspects of cancer biology [36]. Obese conditions promote E2 synthesis, and E2 is shown to enhance LIF expression in the uterus [37]. Recently, a first-in-class inhibitor of LIFR, EC359, was developed and shown to block LIF/LIFR signaling. In this study, we examined the utility of EC359 in blocking obesity-mediated signaling that occurs via the LIF/LIFR axis. Using in vitro cell-based assays and in vivo xenograft assays, we demonstrated that EC359 substantially reduced obesity-mediated EEC progression.

## 5. Conclusions

The global RNA-seq results from this study suggest that obese conditions have the potential to promote activation of IL-6 cytokine and STAT3 signaling pathways in EEC. EEC tumor patients with a high BMI exhibited elevated levels of LIF expression. Using multiple in vitro assays and EEC model cells, this study provided evidence that obese conditions activate STAT3 signaling via the LIF/LIFR axis. Our xenograft studies confirmed that LIF/LIFR signaling driven by obese conditions plays a role in the progression of EEC in vivo and blocking LIFR signaling with EC359 represents a novel therapeutic approach.

## Data Availability

All data generated for this study are included within this article. RNA-seq data have been deposited in the GEO database under a GEO accession number (GSE214872).

## Supplementary Materials

The following supporting information can be downloaded at: https://www.mdpi.com/article/10.3390/cancers14215400/s1. Supplementary Table S1: List of primers; Supplementary Table S2: Detailed description of normal and cancerous Tissue microarray. Supplementary Figure S1: Original blots.
