# Supplementary material for: Inhibition of LIFR Blocks Adiposity-Driven Endometrioid Endometrial Cancer Growth
Source: Cancers (Basel). 2022 Nov 2;14(21):5400. doi: 10.3390/cancers14215400 (PMC9657203; doi:10.3390/cancers14215400)

Figure S1: Original western blots for Figure 3C. Cropped sections used as figures in the manuscript are marked as a box.

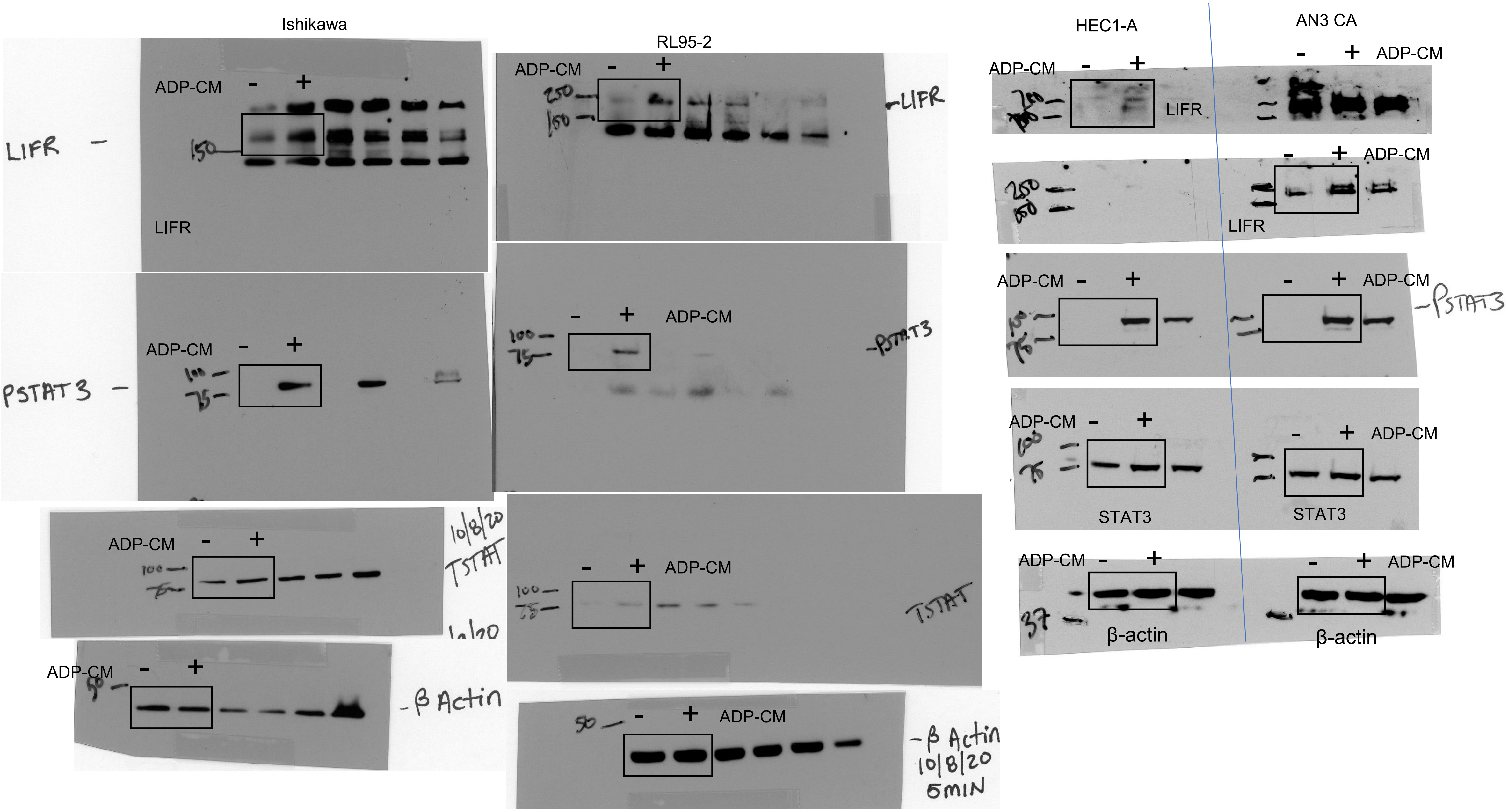

Figure S1: Original western blots for Figure 3 D. Cropped sections used as figures in the manuscript are marked as a box.

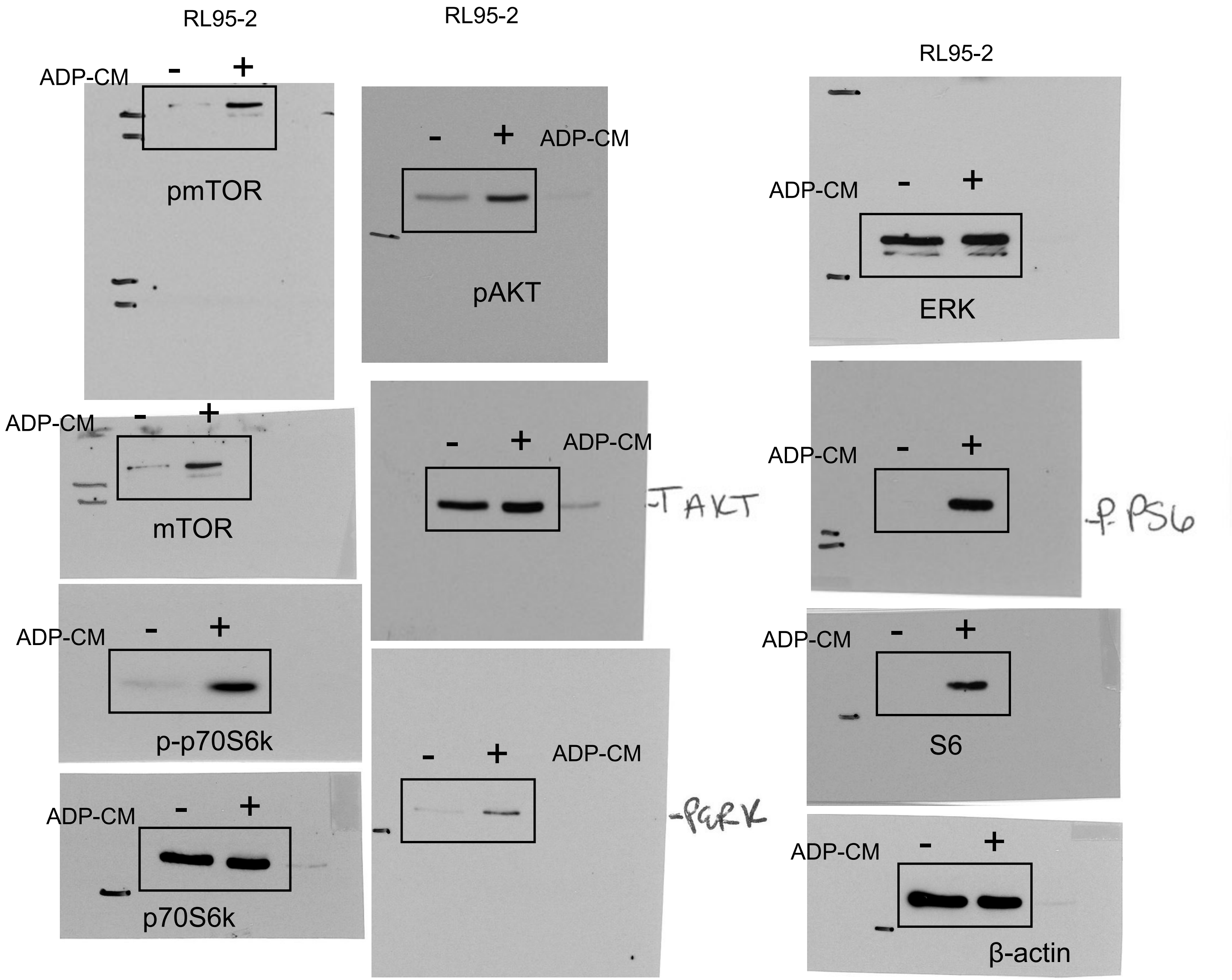

Figure S2: Original western blots for Figure 4F. Cropped sections used as figures in the manuscript are marked as a box.

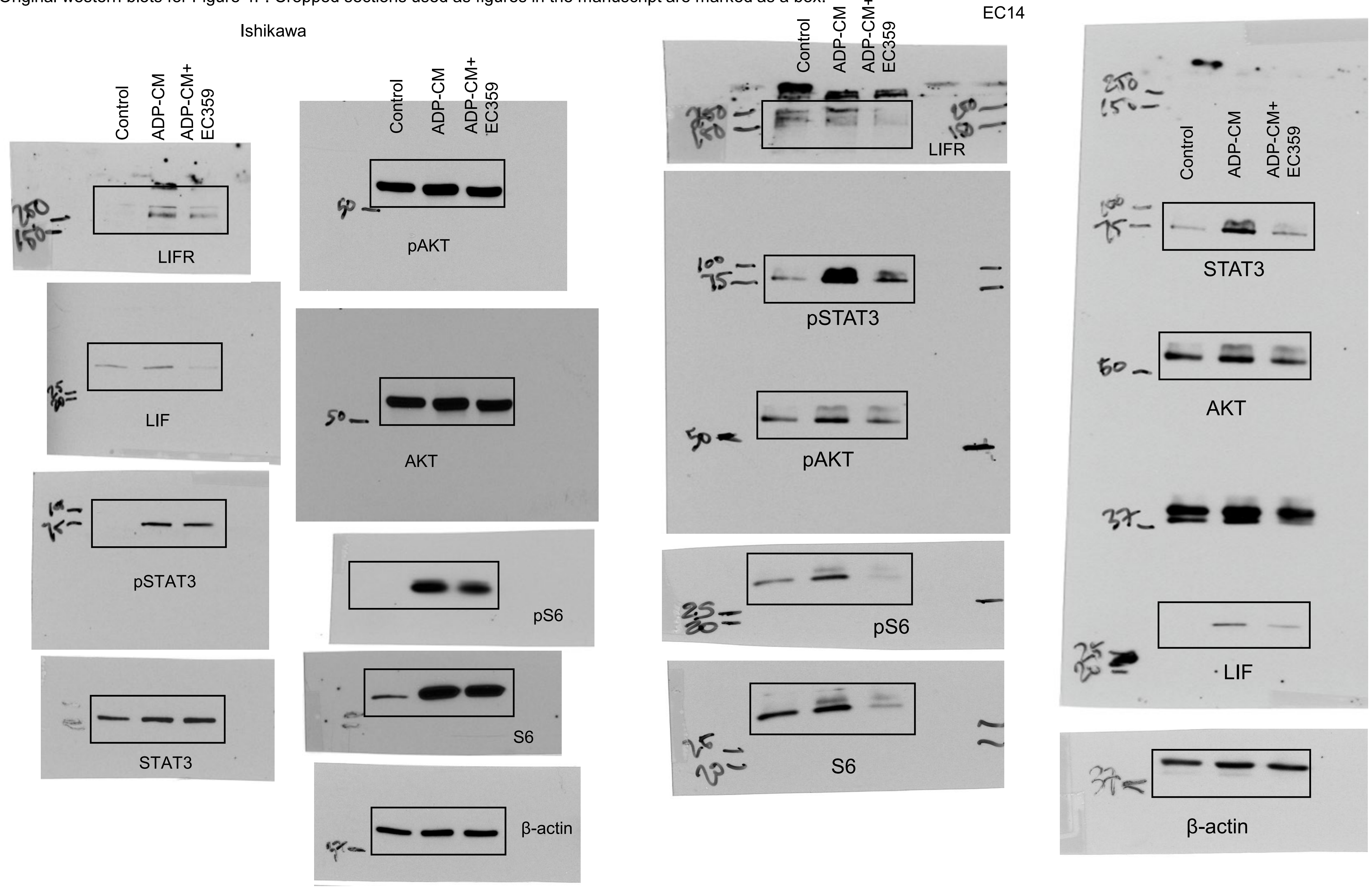

Figure S3: Original western blots for Figure 5A. Cropped sections used as figures in the manuscript are marked as a box.

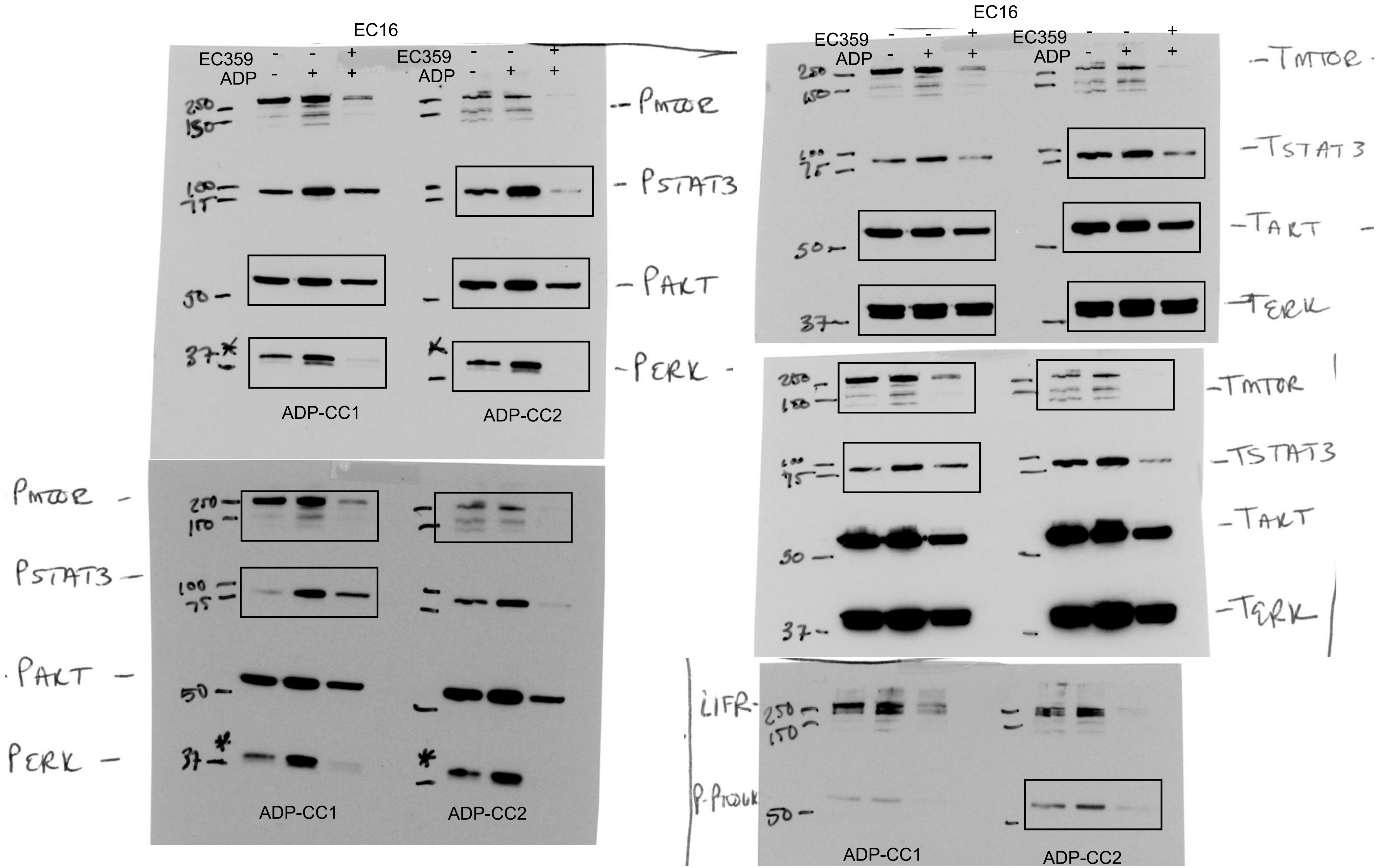

Figure S3: Original western blots for Figure 5A. Cropped sections used as figures in the manuscript are marked as a box.

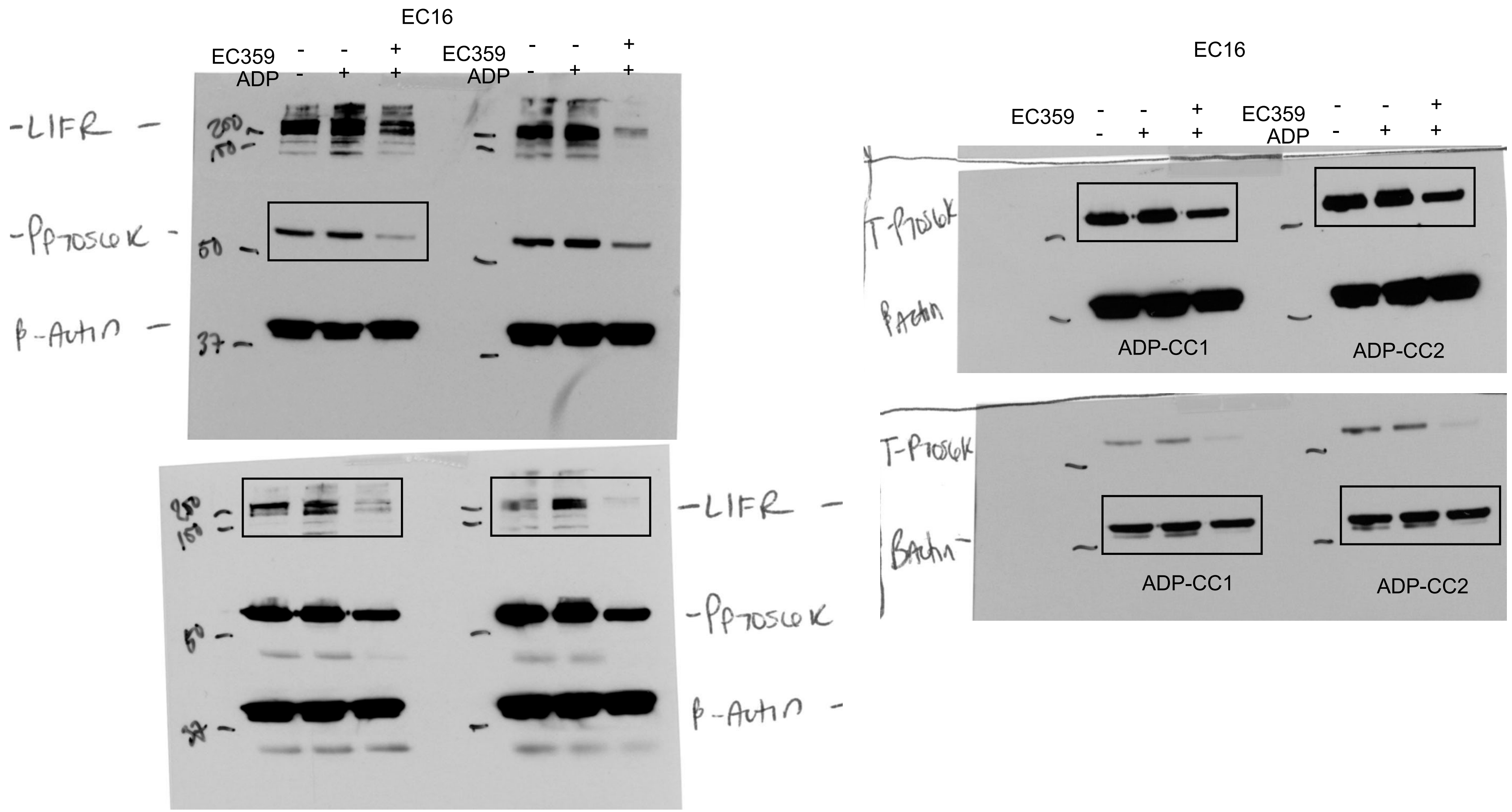

Figure S3: Original western blots for Figure 5 B. Cropped sections used as figures in the manuscript are marked as a box.

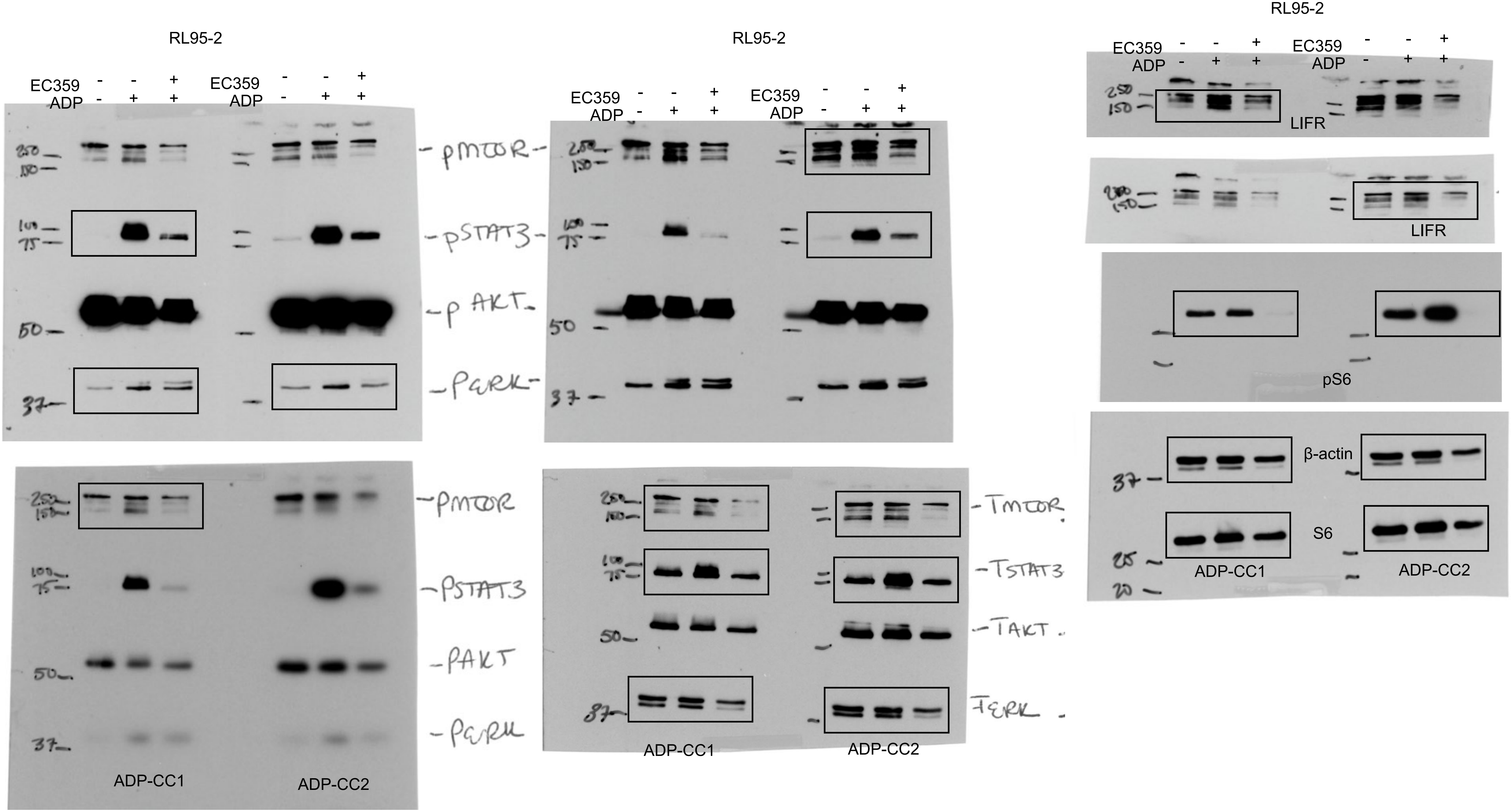

Supplement: Supplementary file 1 [file cancers-14-05400-s001.zip › cancers-1954687-Figure S1 western blots.pdf]
