# Supplementary material for: Inhibition of LIFR Blocks Adiposity-Driven Endometrioid Endometrial Cancer Growth
Source: Cancers (Basel). 2022 Nov 2;14(21):5400. doi: 10.3390/cancers14215400 (PMC9657203; doi:10.3390/cancers14215400)
Supplement: Supplementary file 1 [file cancers-14-05400-s001.zip › cancers-1954687-Supplementary Tables.pdf]

Supplementary Table S1: List of primers

Gene name	Forward Primer	Reverse Primer
GAPDH	TCGACAGTCAGCCGCATCT	CTAGCCTCCCGGGTTTCTCT
LIFR	TGTCAGGCGTTCTCGTCTC	GAGTTGTGTTGTGGGTCACTAA
AKT1	AGCGACGTGGCTATTGTGAAG	GCCATCATTCTTGAGGAGGAAGT
HIF1A	CACCACAGGACAGTACAGGAT	CGTGCTGAATAATACCACTCACA
TGFB1	CAATTCCTGGCGATACCTCAG	GCACAACTCCGGTGACATCAA
ID1	CTGCTCTACGACATGAACGG	GAAGGTCCCTGATGTAGTCGAT
ID2	AGTCCCGTGAGGTCCGTTAG	AGTCGTTCATGTTGTATAGCAGG
ID3	GAGAGGCACTCAGCTTAGCC	TCCTTTTGTCGTTGGAGATGAC
STAT1	ATCAGGCTCAGTCGGGGAATA	TGGTCTCGTGTTCTCTGTTCT
SOX2	TGCGAGCGCTGCACAT	TCATGAGCGTCTTGGTTTTCC
c-MYC	GGCTCCTGGCAAAAGGTCA	CTGCGTAGTTGTGCTGATGT
JUNB	ACGACTCATACACAGCTACGG	GCTCGGTTTCAGGAGTTTGTAGT
TIMP1	CTTCTGCAATTCCGACCTCGT	ACGCTGGTATAAGGTGGTCTG
PTGS2	CTGGCGCTCAGCCATACAG	CGCACTTATACTGGTCAAATCCC
SOCS2	TTAAAAGAGGCACCAGAAGGAAC	AGTCGATCAGATGAACCACACT
HIF1A	CACCACAGGACAGTACAGGAT	CGTGCTGAATAATACCACTCACA
TGFB1	CAATTCCTGGCGATACCTCAG	GCACAACTCCGGTGACATCAA
MCL-1	GTAATAACACCAGTACGGACGG	CCACAAACCCATCCTTGGAAG

Supplementary Table S2: Tissue microarray map

Tumor				
ID	BMI (Kg/m^2)	Histology	Grade	Stage
1	31.4	Endometrioid	1	1
2	31.4	Endometrioid	2	4
3	32.1	Endometrioid	2	3
4	32.1	Endometrioid	2	1
5	32.6	Endometrioid	1	1
6	33	Endometrioid	1	1
7	33.4	Endometrioid	1	7
8	34	Endometrioid	1	1
9	34.7	Endometrioid	1	1
10	35	Endometrioid	2	6
11	36	Endometrioid	2	1
12	36.7	Endometrioid	1	1
13	37.6	Endometrioid	1	1
14	38.9	Endometrioid	1	1
15	39	Endometrioid	1	1
16	39.3	Endometrioid	2	1
17	39.9	Endometrioid	1	1
18	40	Endometrioid	2	1
19	40.6	Endometrioid	1	1
20	40.8	Endometrioid	3	1
21	40.9	Endometrioid	1	1
22	41.1	Endometrioid	1	1
23	41.5	Endometrioid	2	1
24	41.9	Endometrioid	1	1
25	42.5	Endometrioid	1	1
26	42.8	Endometrioid	1	4
27	42.9	Endometrioid	1	1
28	43	Endometrioid	1	1
29	43.4	Endometrioid	1	1
30	43.5	Endometrioid	1	1
31	43.5	Endometrioid	2	7
32	43.6	Endometrioid	1	1
33	44.3	Endometrioid	1	1
34	45.7	Endometrioid	2	1
35	45.9	Endometrioid	1	1

Normal	
ID	BMI (Kg/m^2)
1	30.2
2	32.6
3	34.2
4	34
5	35.3
6	35.3
7	43.3
8	44.2
9	43.8
10	30.1
11	33.3
12	30.9
13	36.8
14	37.5
15	38.5
16	40.9
17	31.86
18	31.6
19	38.9
20	37.6
21	38.92
22	42.5
23	42.6
24	34.5
25	38.7
26	39.4
27	38.4
28	36.9
29	45.1
30	58
31	43.5
32	44.6
33	41.9

36	48.6	Endometrioid	1	1
37	49.1	Endometrioid	1	1
38	49.3	Endometrioid	1	1
39	49.5	Endometrioid	1	1
40	50.6	Endometrioid	2	1
41	51.8	Endometrioid	1	1
42	57.3	Endometrioid	1	1
43	58	Endometrioid	2	4
44	58.4	Endometrioid	1	3
45	61.5	Endometrioid	1	1
46	68	Endometrioid	2	1
